# Impact of positive end-expiratory pressure application on ventriculo-arterial coupling in decompensated left ventricles after cardiac surgery: a non-invasive echocardiographic study

**DOI:** 10.1186/cc13382

**Published:** 2014-03-17

**Authors:** P Bertini, V Simone, R Baldassarri, L Doroni, F Guarracino

**Affiliations:** 1 University Hospital, Pisa, Italy

## Introduction

The management of ICU patients following heart surgery can be hustling when coping with severe left ventricular (LV) dysfunction. Single beat (Sb) measurements of ventriculo- arterial coupling (VAc) can be used by the intensivist when dealing with altered hemodynamic states [1]. LV elastance (Ees) and arterial elastance (Ea) can be measured by trasthoracic echocardiography (TTE) in a Sb fashion [2], so allowing quick assessment of VAc. PEEP application is common practice in the ICU but can have hemodynamic consequences and lead to instability. Speckle tracking analysis by TTE has been recently reported to help titrating PEEP in critically ill patients [3]. However, this can be demanding and require specific ultrasound tools. In this study we aimed to assess whether standard TTE can be useful in evaluating the effect of respiratory treatment with PEEP on cardiovascular efficiency by measuring VAc after coronary artery bypass surgery (CABG).

## Methods

TTE was performed before anesthesia induction and upon ICU arrival in 15 patients with preoperative diagnosis of LV dysfunction defined as an EF <35%. In-ICU measurements of Ees, Ea and VAc were taken at different steps of PEEP application lasting 3 minutes as follows: 0 cmH_2_O PEEP (ZEEP), 5 cmH_2_O PEEP, 10 cmH_2_O PEEP and 15 cmH_2_O PEEP. TTE and hemodynamic parameters were recorded and analyzed.

## Results

All patients were uncoupled preoperatively (VAc >1.31, average VAc (AVAc) = 1.56) before anesthesia induction and showed worsened uncoupling postoperatively at ZEEP. PEEP application altered VAc by modifying both Ees and Ea at all steps and all patients showed further uncoupling at any level of PEEP application (AVAc at ZEEP = 1.97, at 5 cmH_2_O PEEP = 1.61, at 10 cmH_2_O PEEP = 1.87, at 15 cmH_2_O PEEP = 2.23) A PEEP of 5 cmH_2_O provided the more favorable VAc.

## Conclusion

Sb evaluation of Ea/Ees shows that following CABG the patients with depressed LV remain uncoupled, and that in such patients the application of PEEP leads to further decoupling. In this preliminary experience, 5 cmH_2_O PEEP seems to be the most appropriate in preventing further worsening of the VAc. A large RCT is needed to draw conclusions on the PEEP effect on VAc following CABG in a depressed heart.
